# An ultra-portable, self-contained point-of-care nucleic acid amplification test for diagnosis of active COVID-19 infection

**DOI:** 10.1038/s41598-021-94652-0

**Published:** 2021-07-26

**Authors:** Hao Deng, Asanka Jayawardena, Jianxiong Chan, Sher Maine Tan, Tuncay Alan, Patrick Kwan

**Affiliations:** 1 Department of Neuroscience, Central Clinical School, Monash University, Melbourne, VIC 3004 Australia; 2 Department of Mechanical and Aerospace Engineering, Monash University, Clayton, VIC 3800 Australia

**Keywords:** Biological techniques, Biotechnology, Molecular biology

## Abstract

There is currently a high level of demand for rapid COVID-19 tests, that can detect the onset of the disease at point of care settings. We have developed an ultra-portable, self-contained, point-of-care nucleic acid amplification test for diagnosis of active COVID-19 infection, based on the principle of loop mediated isothermal amplification (LAMP). The LAMP assay is 100% sensitive and specific to detect a minimum of 300 RNA copies/reaction of SARS-CoV-2. All of the required sample transportation, lysing and amplification steps are performed in a standalone disposable cartridge, which is controlled by a battery operated, pocket size (6x9x4cm^3^) unit. The test is easy to operate and does not require skilled personnel. The total time from sample to answer is approximately 35 min; a colorimetric readout indicates positive or negative results. This portable diagnostic platform has significant potential for rapid and effective testing in community settings. This will accelerate clinical decision making, in terms of effective triage and timely therapeutic and infection control interventions.

## Introduction

The novel coronavirus disease 2019 (COVID-19) has resulted in a global pandemic. The virus responsible for this is the severe acute respiratory syndrome coronavirus 2 (SARS-CoV-2). It is a positive-sense single-stranded RNA virus which primarily spreads between people through close contact and via respiratory droplets. Viral testing, contact tracing and isolation are widely used public health strategies to restrict the spread of the virus^1^. There is currently a huge demand for COVID-19 testing. At the time of writing more than 2 million tests for active COVID-19 infection are being carried out in the United States every day^2^. Even when vaccines are available it is likely that a high level of testing will remain for public health surveillance^3,4^. It is therefore important to have affordable and easy to use, or even portable technologies for COVID-19 testing in addition to the current gold standard of testing^5^. Indeed, the WHO expert group has identified testing as the first of eight research priorities in response to the COVID-19 outbreak^6^. Point-of-care tests (POCT) can help accelerate clinical decision making, enable effective triage and timely therapeutic and infection control interventions^7^. This will alleviate pressure on overburdened centralized laboratories and allow testing in community settings.

There are currently two main types of tests for COVID-19: serology-based antibody tests and molecular tests. Antibody tests detect the presence of the antibodies (IgM and/or IgG) to SARS-CoV-2 virus in peripheral blood produced by the person as the result of the infection. Therefore, these tests do not accurately represent active infection^8^. In comparison, molecular tests detect viral materials directly in a specimen taken from an individual to indicate active infection. Some molecular tests detect specific proteins from the virus and are known as antigen tests while most molecular tests are based on nucleic acid amplification (nucleic acid amplification tests, NAAT), using either polymerase chain reaction (PCR) or loop mediated isothermal amplification (LAMP)^9^. While antigen tests have the potential for rapid diagnosis, their sensitivity is generally lower compared with the NAAT^10–12^. The current gold standard of testing for SARS-CoV-2 virus is laboratory based NAAT known as quantitative reverse transcription polymerase chain reaction (RT-qPCR)^13^. This test is performed using a relatively specialized expensive equipment (qPCR machine) in a specialized pathology laboratory.

LAMP is a good candidate for NAAT POCT development as it is relatively rapid, simple to operate and requires simple equipment. LAMP uses six primers that identify eight regions on the viral RNA. The reaction runs on a single temperature^14^. To date, the FDA has approved 173 NAATs for COVID-19 diagnosis, of which six are LAMP-based^15^. The key advantage of LAMP-based tests over PCR-based counterparts is their shorter sample-to-result time. However, the actual testing time may be longer than reported after including the time taken for the sample lysing and preparation steps. Currently, all but one of these LAMP-based tests require the use of large, non-portable accompanying equipment for detection or sample lysing^16^. Therefore, most of these tests are near-POCT NAATs as the requirement of specialized laboratory instruments, separate RNA extraction step and large benchtop readout units operated by highly trained individuals would ultimately limit their use at a point-of-care setting. There is an urgent need for point-of-care NAATs that are rapid, simple and portable, and can be operated by minimally trained individuals.

Here, we describe the prototype of such a test based on reverse transcription (RT)-LAMP amplification. It is carried out using a self-contained device and does not require specialized laboratory instruments, a separate RNA extraction step or large benchtop readout units. Instead, it is designed to integrate RNA extraction on the disposable cartridge through boiling of diluted samples. This method has been shown to be effective for the extraction of RNA from oropharyngeal swab samples for the detection of other viral targets^17^. This pocket-size device is portable and simple to operate and can be run using batteries or a power bank. The direct sample to result time is approximately 35 min. No additional sample transfer steps are required as the nasopharyngeal swab is loaded directly into the fully sealed disposable cartridge, allowing safe testing outside a standard laboratory setting. The device is adaptable to test for other respiratory viruses by simply changing the LAMP primers.

## Results and discussion

### Design overview

**Figure 1:**
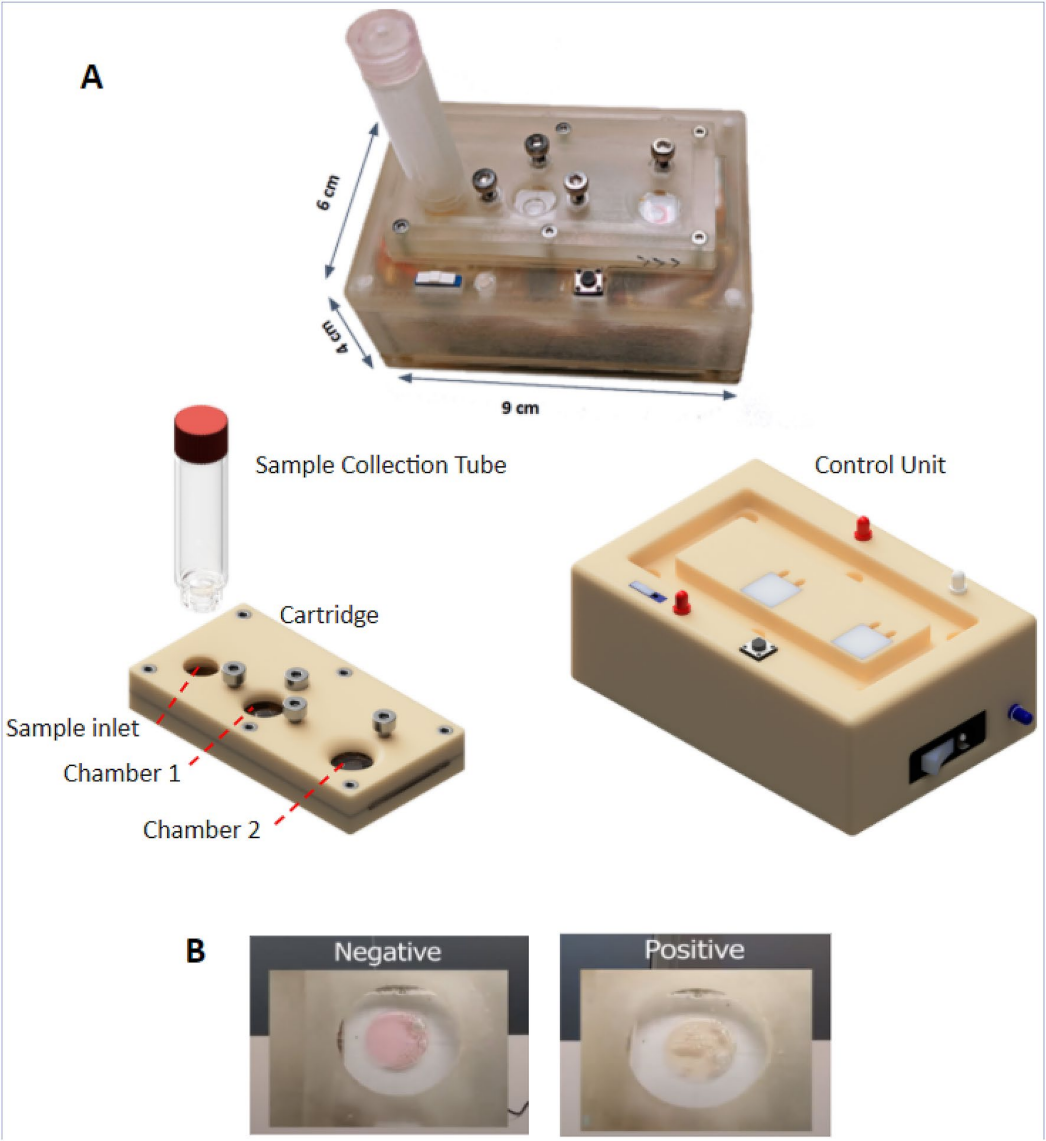
Overall design of the device. (**A**) A photograph of the actual device and a rendered image of its various components which include a collection tube, a disposable cartridge and a reusable control unit. (**B**) The readout of the result in Chamber 2. Pink indicates negative and yellow indicates positive result.

The device comprises 3 main components: a disposable sample collection tube, a disposable cartridge, and a reusable control unit (Fig. 1A).

To perform the test, a nasopharyngeal swab or saliva sample is collected and placed inside the collection tube which contains deionized (DI) water. The tube is then screwed onto the cartridge which has a lysing chamber for sample lysing/RNA extraction (Chamber 1), and a preloaded reaction chamber for LAMP reaction (Chamber 2). The reusable control unit contains heating pads that control the temperature and time of heating for RNA extraction and LAMP reaction in the chambers on the cartridge.

The sample is drawn into Chamber 1 where it is heated at 95 °C for 5 min. It is then moved to Chamber 2, which contains the LAMP mastermix, and where it is heated to 60 °C for 30 min. In the presence of SARS-CoV-2 virus the color of the reagent in Chamber 2 changes from pink to yellow (Fig. 1B). In contrast, if the sample does not contain SARS-CoV-2, the color of the reagent will remain pink.

### SARS-CoV-2 LAMP assays

**Figure 2:**
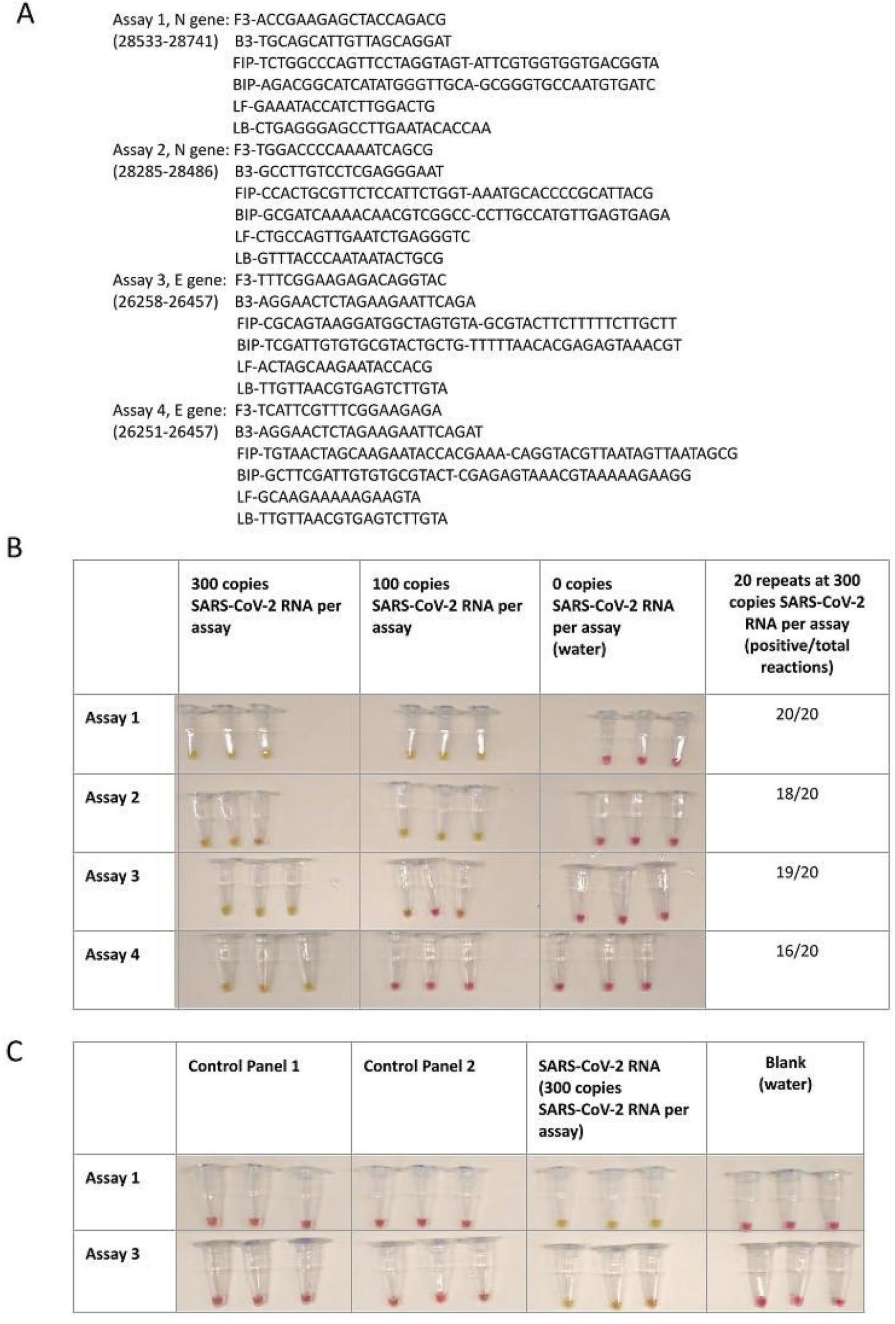
LAMP assays design and validation. (**A**) Primer sequences of LAMP assays. (**B**) Limit of detection of the assays. (**C**) Cross-reactivity validation using NATtrolTM Respiratory Panel 2 (RP2) Controls. Pink color reaction indicates negative and yellow color indicates positive result. Nuclease free water was used in blank samples.

We designed four different sets of LAMP assays, two targeting the N gene and the other two targeting the E gene (Fig. 2A). Sequence alignment indicated that all 4 sets matched against publicly available SARS-CoV-2 sequences (GenBank: MN908947.3) and would detect the standard SAR-CoV 2 strain as well as the later emerged variants such as the UK variants (B.1.1.7, mutation outside primer area) (Fig. 2A)^18^.

The limit of detection (LOD) of these assays was first established using control RNA containing both E gene and N gene sequences of SARS-CoV-2. The assay that gave best performance was used in the device subsequently. The LOD was determined according to U.S. Food and Drug Administration (FDA) guidelines (emergency use diagnostics) and was defined as the lowest concentration of viral detection at which at least 19 out of 20 replicates are positive^19^.

LAMP reactions were performed using colorimetric LAMP mastermix where a positive reaction will turn the reagent from pink to yellow while negative reaction will remain pink. The LOD was determined in two steps. In the first step, LAMP reactions were performed in triplicates on all four sets of assays with spiked RNA controls at concentration of 300 copies, 100 copies or 0 copies of RNA per reaction. As shown in Fig. 2B, all four sets of the assays were able to detect 300 copies of spiked SARS-CoV-2 RNA per reaction, but only two sets (Assay 1, Assay 2) were able to detect 100 copies per reaction. Therefore, 300 copies of spiked SARS-CoV-2 RNA per reaction were used in the second step. In this step, 20 replicates of LAMP reactions were performed for each assay. Assays 1 and 3 were able to achieve at least 19 of 20 positive reactions, while Assays 2 and 4 achieved 18 and 16 positive reactions, respectively. In accordance with the FDA guidelines these results demonstrated that the LOD of Assays 1 and 3 was 300 copies of SARS-CoV-2 RNA per reaction, while Assays 2 and 4 did not meet the FDA guidelines in establishing the LOD.

After determining the LOD we performed further validation (cross-reactivity) on Assays 1 and 3. The cross-reactivity tests were performed on a range of common respiratory viral pathogens using the NATtrolTM Respiratory Panel 2 (RP2) Controls, which contain 22 common respiratory flora and other viral pathogens ranging from Adenoviruses, Coronaviruses and Influenza viruses (see supplementary Table 1). This panel contains 2 control sets (Control 1 and Control 2) which covers all the viral pathogens in the panel. As shown in Fig. 2C, both Assays 1 and 3 did not react to RP2 controls but only showed positive reactions with SARS-CoV-2 positive control. This suggested that both assays are specific to SARS-CoV-2 RNA. Assay 1 targets the N gene, which has been a traditionally preferred target of coronavirus because it is abundantly expressed^20–23^. Thus, given its greater sensitivity (100%), Assay 1 was selected to be used in the device.

### Sample collection tube

**Figure 3:**
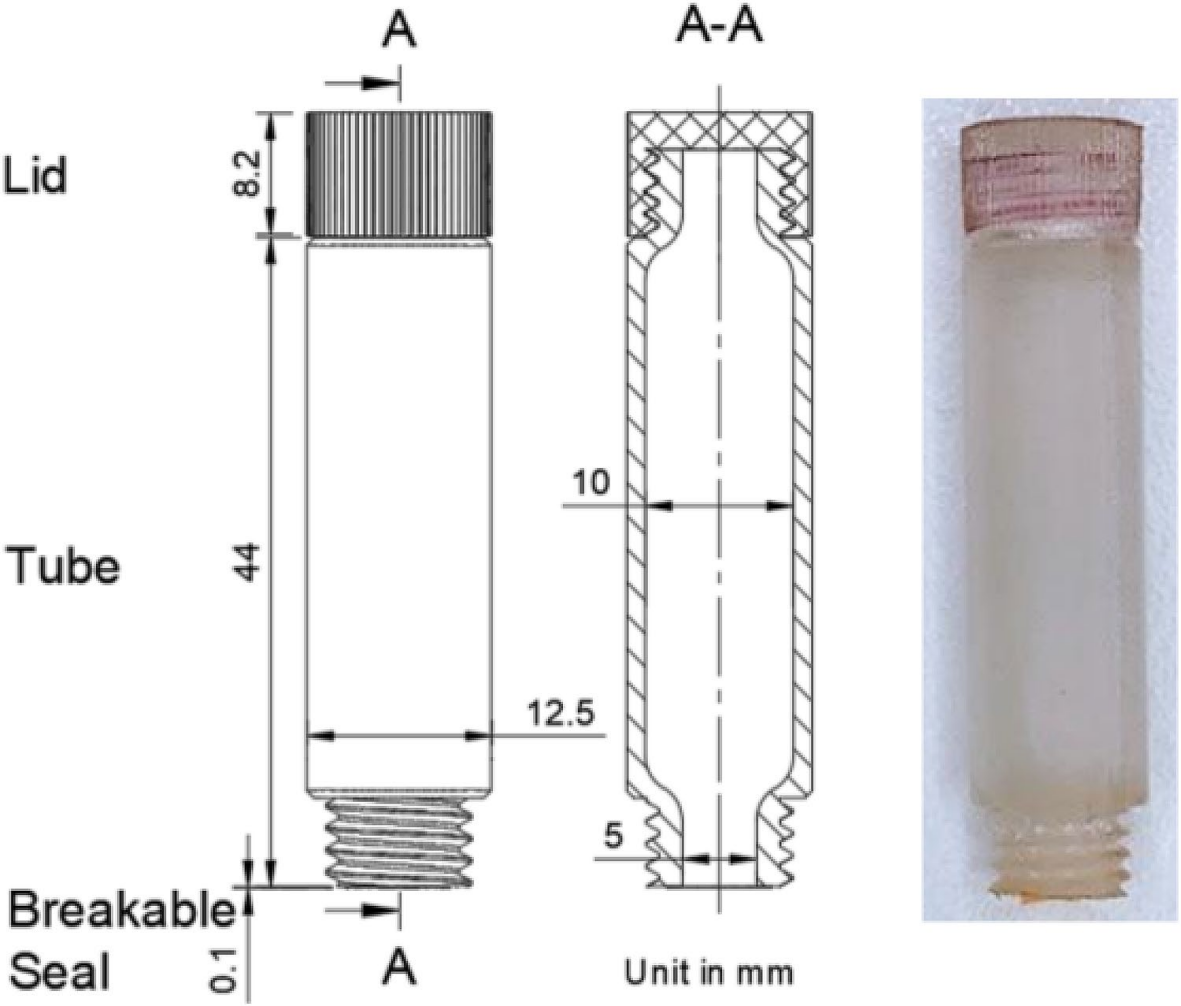
Design drawing and photograph of the sample collection tube. Two openings at different ends of the tube, one end for sample entering and the other connects to the cartridge.

The sample collection tube (Fig. 3) is a cylindrical tube, which is partially filled with DI water. The tube has physical dimensions of 12.5 mm (Diameter, D) × 52.3 mm (Length, L) and is additively manufactured using RGD720 photopolymer. It is designed to collect the sample and introduce it to the disposable cartridge while maintaining the device as a closed system. This is made possible with two threaded openings at different ends of the tube. The sample or swab is placed in the tube via the opening at the top, which is covered with a lid after collection. The other opening that is to be connected to the disposable cartridge, is sealed with a polyamide adhesive film. This film would be punctured by the needle on the disposable cartridge upon connection, allowing the collected sample to enter the cartridge.

### Main cartridge

**Figure 4:**
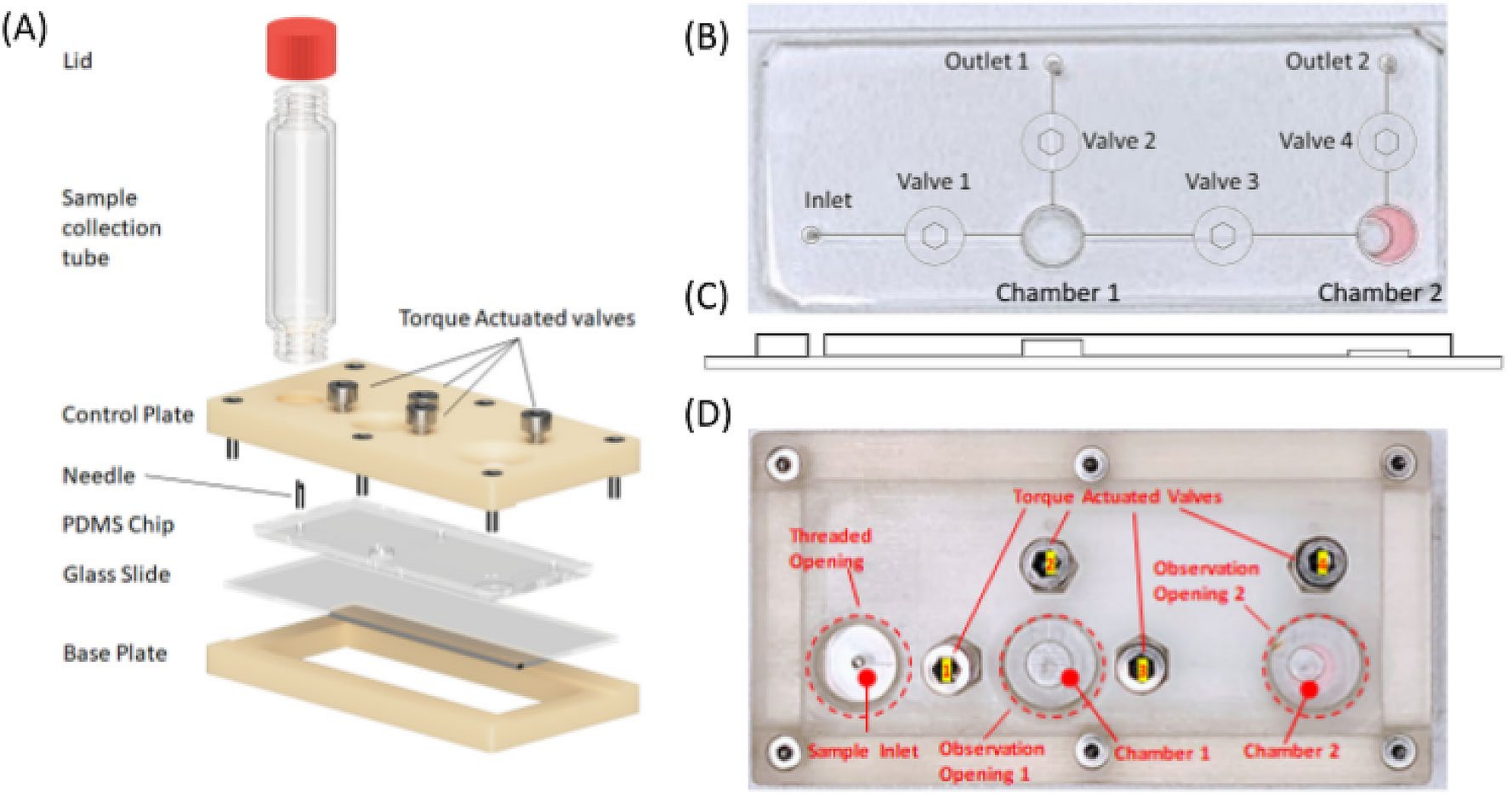
Disposable cartridge design. (**A**) Exploded view of the cartridge-sample collection tube assembly. (**B**) Microfluidic schematic overlapped with a photograph of the microfluidic chip, associated with (**C**) a sectioned view of the chip. (**D**) Top view of the assembled main cartridge.

The main cartridge component (Fig. 4) is a 73 mm (Length, L) × 38 mm (Width, W) × 10.9 mm (Height, H) rectangular block consisting of a 2-mm-thick microfluidic chip sandwiched between the additively manufactured control plate and the base plate.

### Microfluidic chip

The microfluidic chip has one inlet and two outlets, and micro-channels connecting the sample inlet tube with the Chamber 1 and Chamber 2 (Fig. 4B,C). It is fabricated from polydimethylsiloxane (PDMS) with a single soft-lithography step using a 3D printed mold, and is plasma bonded onto a glass slide. The inlet, to which the sample collection tube connects, has a hollow needle assembled with a sharp tip pointing upwards (Fig. 4A,D). This breaks the membrane seal on the sample collection tube upon contact.

Both Chambers 1 and 2 are compressible and they function as press pumps to move the sample between the chambers. Chamber 1 (sample lysing/RNA extraction) is 5.2 mm (D) × 1.5 mm (H). It draws samples from the connected sample collection tube for sample lysing. Chamber 2 measures 5.2 mm (D) × 0.6 mm (H) and is prefilled with 8 µl of LAMP master mix. It draws in approximately 5 µl of processed sample from Chamber 1 for LAMP reaction. Connecting the chambers are micro-channels with dimensions of 150 µm (W) × 100 µm (H). The flow through the channels is controlled by the torque actuated valves on the control plate.

### Control plate

The control plate is additively manufactured from RGD720 photopolymer. It is inserted with four nuts and bolts which act as torque actuated valves to manipulate the opening and closing of the microchannel. This is done by applying pressure on the top surface of the PDMS chip, to control the flow. The surface of the plate consists of three openings: one threaded opening that aligns with the chip inlet to mount the sample collection tube, and two observation and manipulation openings on top of Chambers 1 and 2.

### Mechanism of torque actuated valves and press pumps

Over the past decade, there has been a growing interest in manually actuated liquid pumping systems for repeated sample transfer in microfluidic systems. Numerous elegant designs were demonstrated to manipulate fluids using press valves^24–27^. However, in the majority of these devices, the operational simplicity is often ensured at the expense of fabrication complexity using a combination of soft lithography, injection molding processes, and alignment processes. Here, considering the limited number of sample transfer steps that are required, we use a simpler approach, which can easily be produced with a single soft lithography step, as described in the “Materials and methods” section.

**Figure 5:**
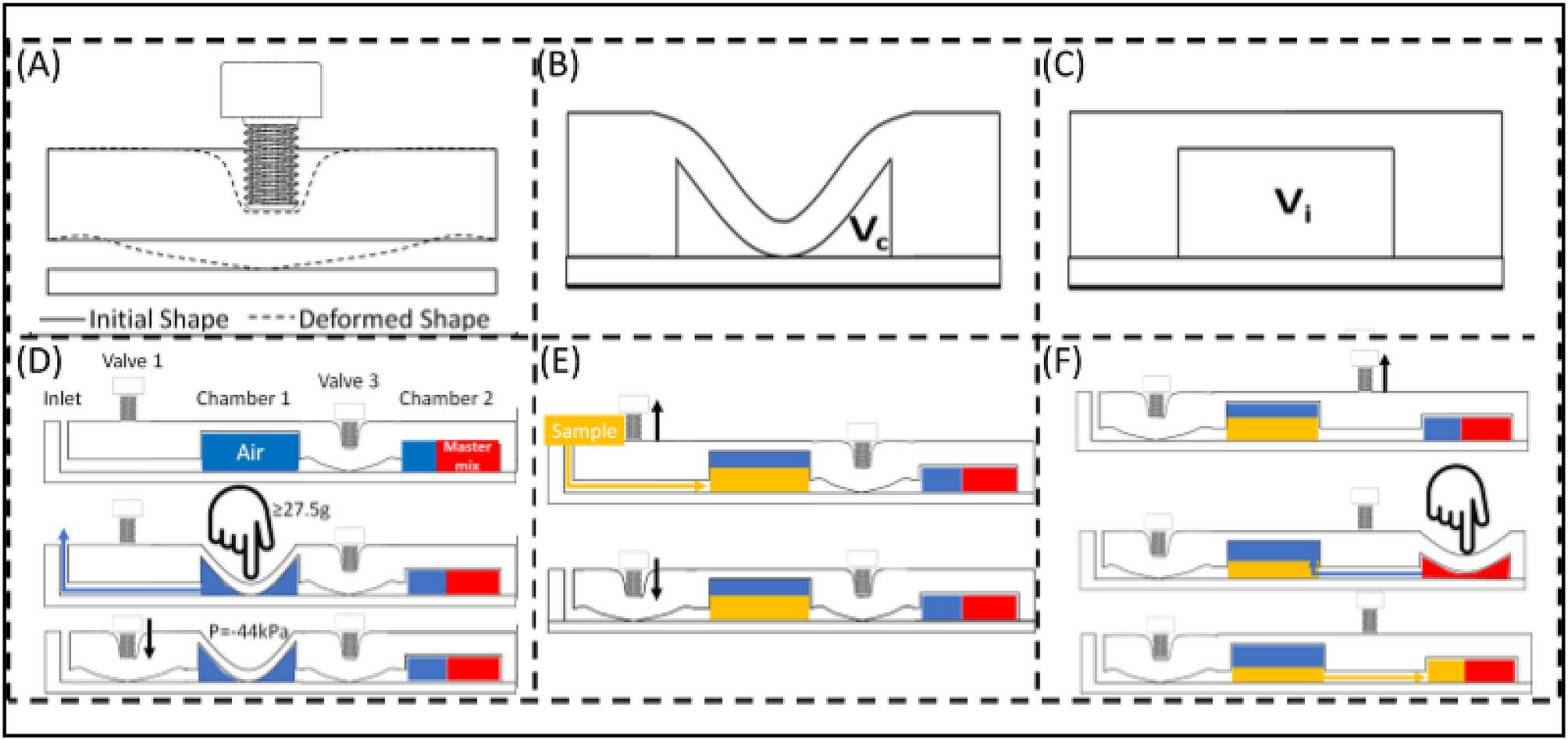
Pumping and Valving mechanism. (**A**) Schematic for the valve mechanism. (**B**) Drawing of a compressed chamber, and (**C**) an uncompressed chamber. (**D**) Process for generating vacuum in Chamber 1. (**E**) Process for loading Chamber 1. (**E**) Process for loading Chamber 2.

In our design, the two compressible chambers on the chip and the torque actuated valves on the control plate are used together to transport and mix small volumes of samples on demand. The mechanisms are illustrated in Fig. 5. The flow between the inlet and the different chambers is controlled by manually tightening and loosening the torque actuated valves. Tightening the torque actuated valves exerts downward pressure on the top surface of the PDMS microchannel. This collapses the channel and blocks the flow. The channel reverts to its original shape when the Valve is opened. The channel connects the different regions of the chip, allowing fluid to move between different regions (Fig. 5A).

Similarly, the flow direction can be modified by pressing on the deformable chambers and locally tuning the pressure differences. The chamber geometry was optimized such that applying only a 0.27 N (27.5 g) load on the chamber ceiling shrinks their volume by 45%, resulting in a − 45 kPa negative pressure environment (Fig. 5B,C, Figure S1, see supplementary information for more detail). As detailed below, and demonstrated in Video S1, this negative pressure is sufficient to rapidly draw up to 14 μl of sample to the chamber in 7 s.

To allow rapid sample transfer from the inlet to the channel, a temporary vacuum environment is generated in Chamber 1 (Fig. 5D). Pressing on Chamber 1, while Valve 2 is open (and all other valves are closed) deforms the chamber ceiling and displaces any air trapped in Chamber 1 towards Outlet 1. Closing Valve 2 ensures a negative pressure in Chamber 1. This can be sustained unless any of the valves are loosened.

After inserting the sample collection tube onto the cartridge at the start of the operation, the user would open Valve 1. This connects the chamber to an atmospheric pressure and converts the negative pressure in the chamber into kinetic energy that drives the sample to flow into the chamber. Valve1 is then closed to prevent sample escaping from Chamber 1 during the lysing step (Fig. 5E).

To transfer the processed sample from Chamber 1 to Chamber 2, the user would open Valve 3 which connects Chambers 1 and 2, then press on Chamber 2 to transfer the 5 µl air bubble into Chamber 1. This action prepares Chamber 2 to generate negative pressure and pressurizes Chamber 1. When the pressure on top of Chamber 2 is released, the pressure difference between two chambers drives 5 µl of lysed sample into Chamber 2. Valve 3 is then closed to prevent sample escaping from Chamber 2 during following steps (Fig. 5F). The operation of the device with SARS-CoV-2 RNA spiked samples is described in “Sample collection tube” section.

### Reusable control unit

**Figure 6:**
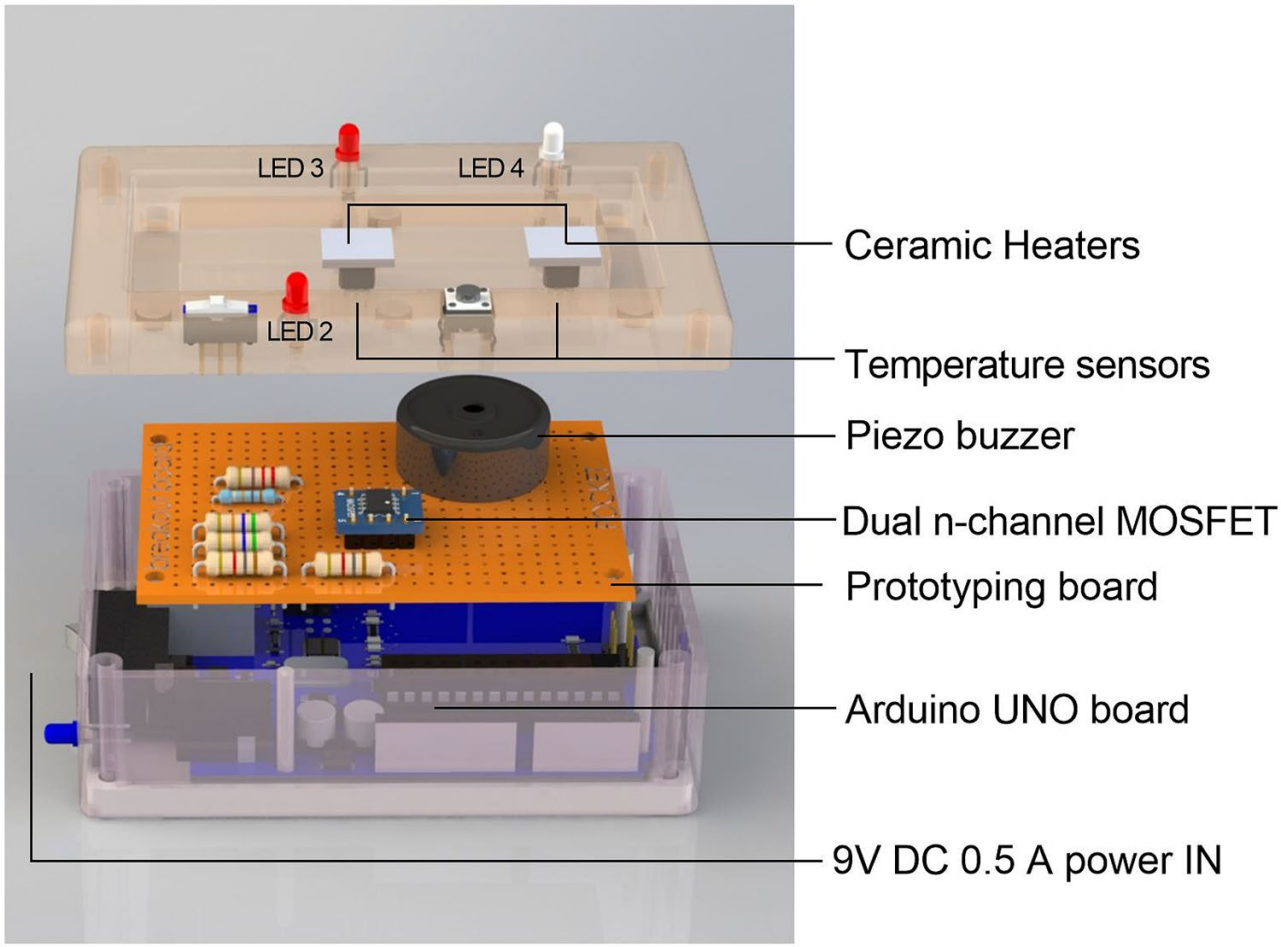
The portable and battery-operated reusable control unit. Exploded view of the microcontroller-based reusable base-unit schematic with the off the shelf (OTS) electronic components.

The control unit (Fig. 6) measures 9 cm (L) × 6 cm (W) × 4 cm (H) and is powered by an external 9 V DC 0.5 A power. The main function of the control unit is to control the temperature on the disposable cartridge to allow cell lysis (Chamber 1) and LAMP reaction (Chamber 2) to take place. The heating is provided by two separate ceramic heaters (1 cm × 1 cm) that align with the positions of the chambers on the cartridge. A programmable microcontroller-based reusable base-unit was developed by using off the shelf (OTS) electronic components and 3D printed enclosure. OTS components were deliberately chosen to meet the objective of rapid prototyping and low cost/power consumption requirements.

Temperature profile and control of the reusable unit were fine tuned to ensure the desired temperature was reached in Chambers 1 and 2. This required precise estimation and consideration of various factors including: (a) Power required to bring the chambers to the set temperature in a given time. (b) Heat loss through air flow, conduction through support structures (glass, PDMS, 3D printed parts), and thermal radiation. (c) Properties of the material to be heated such as state of the materials, dimension and density of the materials to be heated, specific heat, original temperature, final temperature and expected time to reach final temperature. (d) Offsets in temperature sensor: feedback temperature varied from the actual temperature due to heat transfer properties, delays, placement of the sensors and inherited properties/capabilities of the sensors.

**Figure 7:**
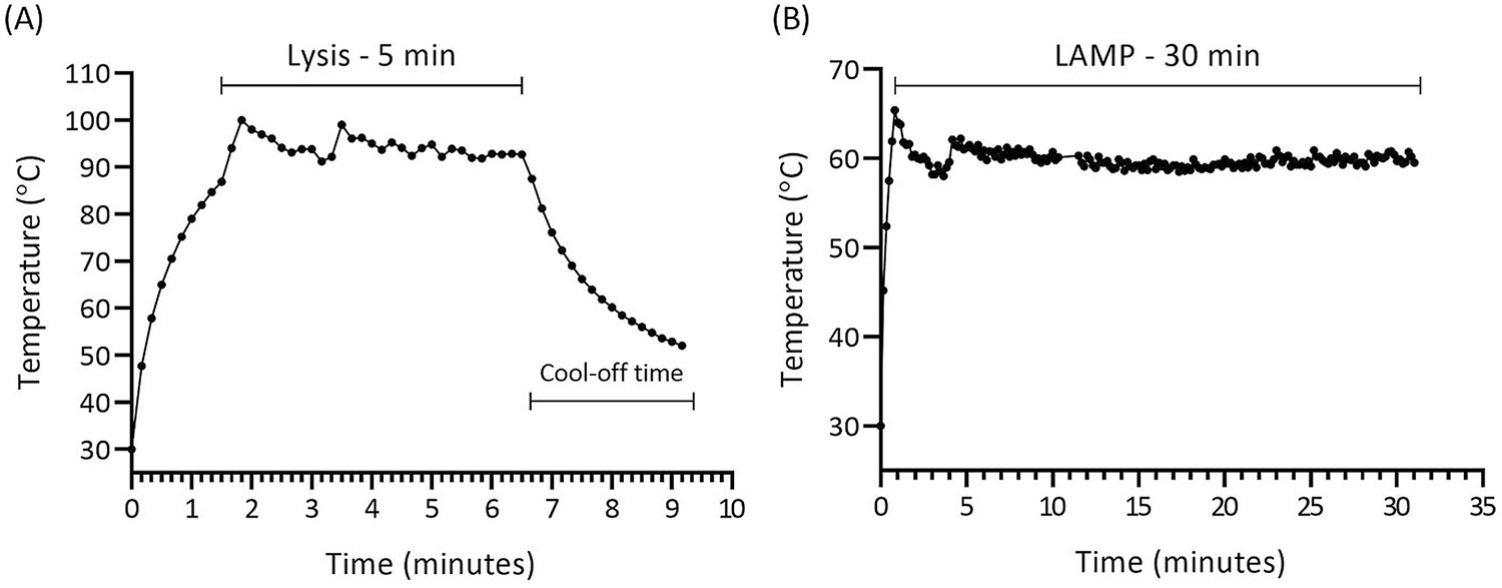
Plots of temperature readings in 10 s intervals. (**A**) Chamber 1 temperature profile showed desired temperature 95 °C with the standard deviation (SD) of 2.5 °C maintained for 5 min. (**B**) Chamber 2 temperature profile showed desired temperature 60 °C with the SD of 0.9 °C maintained for 30 min.

Thermal profiling and temperature control were done with FLIR i7 infrared thermal camera followed by modification of controlling sketch and/or design to fine tune accordingly. Figure 7 shows the graphical representation of the temperature readings for the designated chambers measured every 10 s. It was initially found that ceramic heaters had higher heat-up rate, and heat transfer from heater to glass was quicker than heat transfer to the temperature sensor. Therefore, the feedback temperature from the sensors was not accurate during the warm-up step, and introduced overheating. Also, it was realized that implementation of the standard proportional–integral–derivative (PID)control algorithm would not be reliable due to the properties of the chosen temperature sensor and heat transfer properties. To mitigate the overshooting and to keep the heating process under control, “Ramp to Setpoint” algorithm was used in the program sketch. The “Ramp to Setpoint” process controls the heating rate during the heating process by shortening and tuning the duty cycle time (on–off time) of the heater. In this regard, the cycle time was shortened to below 1 s. As shown in Fig. 7, the implementation of the tuning algorithm lowered overshooting temperature and shortened the overshooting period (less than 20 s). These resulted in well controlled and homogeneous thermal profiles for the respective chambers. Video recordings for the thermal profiling of the lysis process and LAMP reaction can be found in the supplementary information (Video S2, S3).

### Run on spiked SARS-CoV-2 sample: Step-by-step procedure

**Figure 8:**
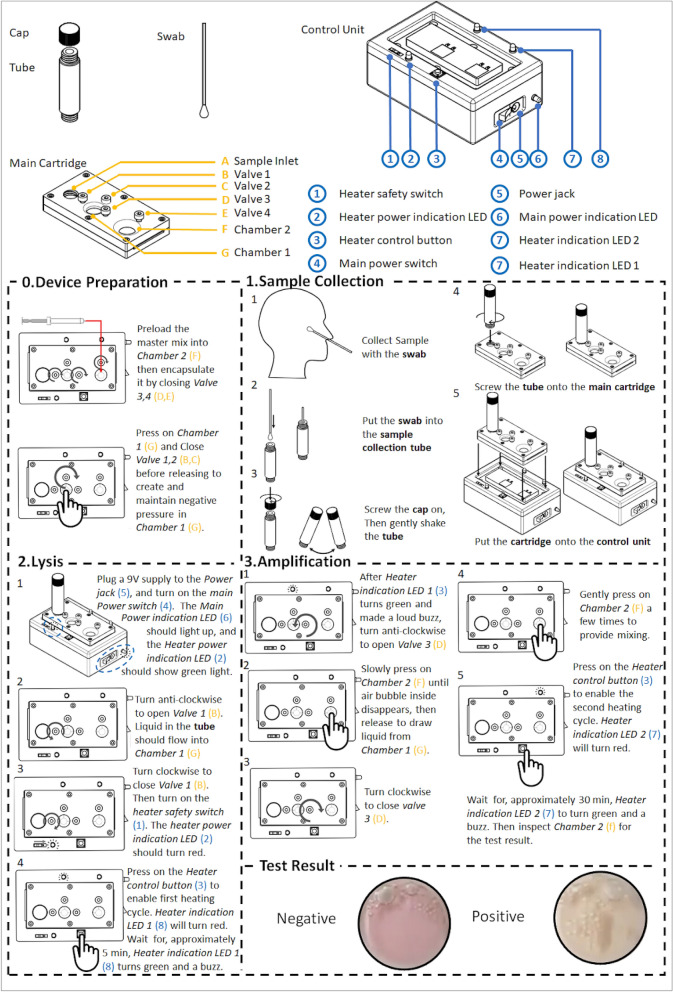
Step-by-step operating procedure of the device.

Details of the operating steps are illustrated in Fig. 8 and in Video S1. For the purpose of prototype testing water spiked with 300 copies of SARS-CoV-2 RNA was used. Before use, 8 µl of LAMP master mix from Assay 1 was introduced via a syringe through Outlet 2 into Chamber 2. Chamber 1 was then pre-compressed, and all valves were configured to a closed state. The sample collection tube was pre-loaded with 1.5 ml DI water as a lysing medium.

In Step 1, the SARS-CoV-2 RNA spiked sample of water was added into the sample collection tube. After closing the lid, gentle shake was applied to ensure samples were mixed in the DI water. The sample collection tube was then connected into the disposable cartridge via a screw in mechanism. This led to the on-chip needle on the disposable cartridge breaking the membrane seal, allowing the sample to flow into the cartridge.

In Step 2, the cartridge was placed on the reusable control unit where the switch was turned to the “on” position. The initiation of the testing steps was indicated by turning the LEDs color to red. Valve 1 was then switched from close state to open state where Chamber 1 returned to its original shape from its pre-vacuumed form. This action drew a small amount of sample from the sample collection tube into Chamber 1. Valve 1 was closed to prevent backflow. In future testing of clinical samples, it is envisaged that RNA would be extracted through thermal lysis as described previously to be an effective method for rapid RNA extraction^17^. The lysis process would be initiated by pushing the tactile switch, which heated up Chamber 1 to 95 °C within 2 min, and the temperature would be maintained at 95 °C ± 2.5 °C for the next 5 min. At the end of the lysis process, 2.5 min of cool-off wait time period was introduced to meet the temperature stability requirement (< 70 °C) of the LAMP reagents. At the end of the lysis process steps, the LED color changed from red to green and the buzzer will start to beep.

In Step 3, Valve 3 was opened to allow flow of the lysed sample from Chamber 1 to Chamber 2. Chamber 2 was pressed to transfer its air bubble to Chamber 1. Upon releasing, Chamber 2 returned to its original shape and drew in 5 µl of the lysed sample. Valve 3 was then closed. Gentle pressing on Chamber 2 effectively mixed the sample with the preloaded LAMP master mix, which had a pink color. The LAMP reaction was initiated by pushing the tactile switch for the second time. The LAMP desired temperature of 60 °C was reached within 1 min, and was maintained at 60 °C ± 0.9 °C for the next 30 min. At the end of the 30 min, the LED changed from red to green and the buzzer started to beep continuously to indicate the completion of the LAMP reaction.

The result was directly viewed from the Chamber 2. The LAMP master mix solution changed from pink to yellow, indicating a positive result. The color would remain pink in a negative test.

### General note

The device developed has the potential to complement the current gold standard COVID-19 NAATs based on RT-qPCR. As the device may be used at the point-of-care it will help alleviate pressure on overburdened centralized laboratories. This will accelerate clinical decision making, enabling timely therapeutic and infection control interventions. Importantly, the device opens up the possibility of bringing sensitive NAATs to places where their use is traditionally not feasible, including remote and non-medical settings (e.g. borders, mass gathering venues). This may assist the society to function in a “COVID-normal” state.

Given the highly adaptable nature of this device, future possible mutations of SARS-CoV-2 can be detected by simply modifying the primer set in the LAMP assay. Furthermore, different disposable cartridges targeting other respiratory pathogens can be developed based on this system while maintaining the reusable control unit. This design makes the device highly versatile beyond COVID-19 diagnosis.

A number of improvements can be made to the current prototype. Firstly, the contrast in color between a positive and negative test may be improved by adding a blue-based dye to the LAMP mastermix, or by applying a filter over Chamber 2. These modifications will convert pink/yellow to purple/brown which may be more easily visualized. Secondly, if simultaneously testing for multiple samples is required, the components in the control unit can be substituted with microcontroller board, printed circuit board and heater to accommodate the heating capacity of multiple cartridges. Thirdly, the testing procedures may be automated by integrating electromechanical actuators onto the device to perform the sample transfer steps that are currently operated manually. In addition, it is worth noting that thermal lysis generally results in some level of RNA degradation, which may affect the LOD of this protoype^28^. Future testing on clinical samples with known viral counts will be essential for a more representative LOD of this device.

## Conclusion

We have developed an ultra-portable, self-contained POCT prototype for diagnosis of active COVID-19 infection. This is a standalone device which can detect SARS-CoV-2 in approximately 35 min. The device can be adapted to test for other respiratory pathogens. This rapid and miniaturized new diagnostic platform has the potential to enable more timely infection control interventions.

## Materials and methods

### LAMP reaction

LAMP reaction contains 1XWarmStart® Colorimetric LAMP Master Mix (New England Biolabs, Ipswich, Massachusetts), 0.2 µM forward and reverse primers (F3 and B3), 1.6 µM inner primers (FIP and BIP) and 0.4 µM Loop primes (LF and LB). All primers used were desalted grade (Integrated DNA Technologies, Coralville, USA). In each reaction, a template volume of 5 μL was added and reaction was performed at 60 °C for 30 min. For LOD test, positive RNA control (Twist Bioscience, San Francisco, USA) was diluted to either 100 RNA copies or 300 RNA copies per 5 μL. For cross reactivity test, 5 μL of each NATtrolTM Respiratory Panel 2 (RP2) Controls (ZeptoMetrix Corporation New York, USA) was added into the reaction.

### Disposable cartridge

A high-aspect ratio mould for the subsequent soft lithography steps was additively manufactured (RGD 720 multipurpose transparent PolyJet photopolymer, Objet Eden260V PolyJet 3D printer), along with the control plates. To produce the microfluidic chip, a 10:1 PDMS-curing agent mixture (Sylgard 184 silicone elastomer kit) was degassed in a vacuum chamber for 1 h, then poured onto the mould and cured at 70 °C for 8 h. The PDMS was then peeled off the mould and trimmed, using a scalpel blade, to desired size. Channel inlet and outlets were formed with a 1.5 mm biopsy punch. Prior to assembly, the surface of the PDMS was activated by exposing it to an 18 W air/oxygen plasma at 0.6–0.8 torr pressure for 21 s, inside a plasma cleaner (PDC-32G-2, Harrick Plasma). The PDMS channels were then covalently bonded to a glass slide, by bringing them in contact immediately after plasma activation, to enclose the bottom of the channel. This creates the microfluidic chip. 8 µl of master mix was then loaded into the microfluidic chip using a syringe (Becton Dickinson 0.5 ml 29G × 12.7 mm Medical Syringe) before assembling the chip with needle, control plates, and fasteners etc. into the main cartridge. Chamber 1 was pre-vacuumed, by compressing the chamber then closing all valves, before deploying.

### Arduino based reusable unit

The ATmega328P based Arduino UNO REV3 microcontroller board was used for the purpose of easy prototyping. The square-shaped (1 cm × 1 cm), micro metal-ceramic heating (MTH) tablets (R = 20 Ω) were placed under the bottom of both Chamber 1 and Chamber 2 to provide the required thermal heating. The low-power linear active thermistor ICs (MCP9700A) were used as the analog temperature sensors to read the temperature in real time by interfacing with the Arduino Analog Input Pins. The feedback temperatures from the temperature sensors and predefined time intervals were used in the conditional statements in the Arduino program sketch to control and maintain the temperature of chambers by switching on and off the heaters. In this regard, dual n-channel MOSFET (Si4944DY) with the extremely low on-resistance (RDS (on) 0.016Ω @ VGS = 4.5 V) and fast switching properties, was used in the switching circuit. The Arduino Digital I/O pins controlled the switching circuit by controlling the MOSFET gate signal. The micro slide switch (SPDT, latching) and tactile switch (SPST, momentary) were interfaced with the Arduino Digital I/O pins, and used as the user interface to trigger the start of lysis step and LAMP step. Bi-color (green & red) LEDs and external piezo buzzer were interfaced with the Arduino Digital I/O pins, and used as the visual feedback and alarm to indicate the end of the test steps. Other than the MOSFET (Surface-mount technology), resistors, LEDs, thermistor ICs and buzzer were chosen as the through-hole components. Therefore, it was required to solder wires to the breakout PCB board for the interface purpose. The 2.1 mm DC socket with SPST rocker switch was mounted as the power inlet point, which can be used with power banks, battery holders with DC plugs and standard power adapters. The Arduino board was also powered by the same power source via interfacing the VIN pin with power input. The regulated 5 V output on the Arduino board was used as the voltage input for the thermistor ICs. The schematics for the electronics and the list of OTS components can be found in the supplementary information (Figure S2 and Table S2). The enclosure of the reusable base unit was designed to match with the disposable cartridge and to align with the designated chambers with the ceramic heaters. The RGD 720 resin was used in the Object Eden260V 3D printer to perform the 3D printing of the enclosure parts.

For the temperature profiling, FLIR i7 infrared thermal camera (sensitivity ± 0.1 °C) was used with the dummy disposable cartridge on the heaters to observe the behavior. The top PDMS layer of the designated chambers were punctured to accurately profile the temperature inside the chamber. The FLIR i7 camera was clamped and focused on the designated chambers to measure the temperature.

Simplified power selection analysis was performed and the ceramic heating tablets were identified as the main power demanding part of the system. In this regard, 9 V DC 0.5 A was selected as the power supply requirement for the reusable controller setup by considering the final temperature requirements of the heaters and power required to bring the chambers to the set temperature in a given short period of time without occurring an overheating.

### Software

Figures in this manuscript were produced by the authors using the following softwares: Autodesk Fusion 360 version 2.0.10032 (https://www.autodesk.com.au/products/fusion-360), Solidworks 2019 (https://www.solidworks.com), Eeschema KiCad version: (5.1.5)-3 9 (https://www.kicad.org/discover/schematic-capture) and Microsoft Powerpoint version 1808.

## Supplementary Information

Supplementary Information 1.

Supplementary Information 2.

Supplementary Video 1.

Supplementary Video 2.

Supplementary Video 3.

## Abbreviations

µM: Micromolar
3D: Three dimensional
ANFF: Australian National Fabrication Facility
B: Backward Primer
BIP: Backward Inner Primer
COVID-19: Coronavirus disease 2019
D: Diameter
DC: Direct current
DNA: Deoxyribonucleic acid
DI: Deionized
E: Envelope
F: Forward Primer
FDA: U.S Food and Drug Administration
FIP: Forward Inner Primer
H: Height
I/O: Input/output
IC: Integrated circuit
IgG: Immunoglobulin G
IgM: Immunoglobulin M
L: Length
LAMP: Loop Mediated Isothermal Amplification
LF: Forward loop primer
LB: Backward loop primer
MTH: Micro metal-ceramic heating
MOSFET: Metal Oxide Silicon Field Effect Transistor
N: Nucleocapsid
NAAT: Nucleic acid amplification test
OTS: Off the shelf
PCB: Printed circuit board
PCR: Polymerase chain reaction
PDMS: Polydimethylsiloxane
PID: Proportional integral derivative
R: Resistor
RNA: Ribonucleic acid
RP2: Respiratory Panel 2
RT-qPCR: Quantitative reverse transcription polymerase chain reaction
SARS-CoV-2: Severe acute respiratory syndrome coronavirus 2
SD: Standard deviation
SPST: Single pole, single throw.
V: Volt
VGS: Gate source voltage
VIN: Input voltage
W: Width
