# Supplementary material for: An ultra-portable, self-contained point-of-care nucleic acid amplification test for diagnosis of active COVID-19 infection
Source: Sci Rep. 2021 Jul 26;11:15176. doi: 10.1038/s41598-021-94652-0 (PMC8313664; doi:10.1038/s41598-021-94652-0)
Supplement: Supplementary file 1 — Supplementary Information 1. [file 41598_2021_94652_MOESM1_ESM.docx]

**Supporting Information**

An ultra-portable, self-contained point-of-care nucleic acid amplification test for diagnosis of active COVID-19 infection

*Hao Deng,* ^‡,⸸^ *Asanka Jayawardena1,* ^‡,†^ *Jianxiong Chan1,* ^‡^*^,^*^†^ *Sher Maine Tan,*^†^ *Tuncay Alan,* ^*,⸸^ *Patrick Kwan1 ^*^*^,†^

^†^Department of Neuroscience, Central Clinical School, Monash University, Melbourne, VIC 3004, Australia

^⸸^Department of Mechanical and Aerospace Engineering, Monash University, Clayton, VIC 3800, Australia



**Pressure calculation**

Chamber 1’s initial and compressed volume needs to be solved in order to calculate the pressure generated by the pump.

Chamber 1 has an initial volume (V_i_) of 31.86 mm^3^ (Figure S1A) is calculated by, multiplying the base area to its height, using the following formula:

$$V_{i}=\pi\left( \frac{5.2}{2} \right)^{2}\times1.5=31.86 mm^{3}$$

To obtain the compressed Chamber 1 volume: V_c_ (Figure S1B), a finite element analysis was performed, on a 2D axisymmetric model of the chamber, with a load applied uniformly on top of the chamber until the center of the ceiling touches the bottom (Figure S1C). By probing the deformation along the ceiling (Figure S1D) and fitting it with a third order polynomial equation, we obtained a function of the deformation shape in terms of the radio coordinate position:

$$f\left( r \right)=-0.09594r^{3}+0.4896r^{2}-0.0834r$$

The volume of the compressed chamber was then calculated using the shall integration method:

$$V_{c}=2\pi\int_{0}^{2.6} r\times f\left( r \right)dr=17.75 mm^{3}$$

During this process, ΔV of air inside Chamber 1 was displaced toward Outlet 1, where ΔV is calculated as:

$$\Delta V=V_{i}-V_{c}=14.11 mm^{3}$$

When the pressure exerted on the chamber was removed, the chamber returned to its original shape, thereby generating a negative pressure (P_n_). This pressure was calculated, using ideal gas law, as follow:

$$P_{n}=1\left[ atm \right]\times\left( \frac{V_{c}}{V_{i}}-1 \right)=-44.86 kPa$$

**
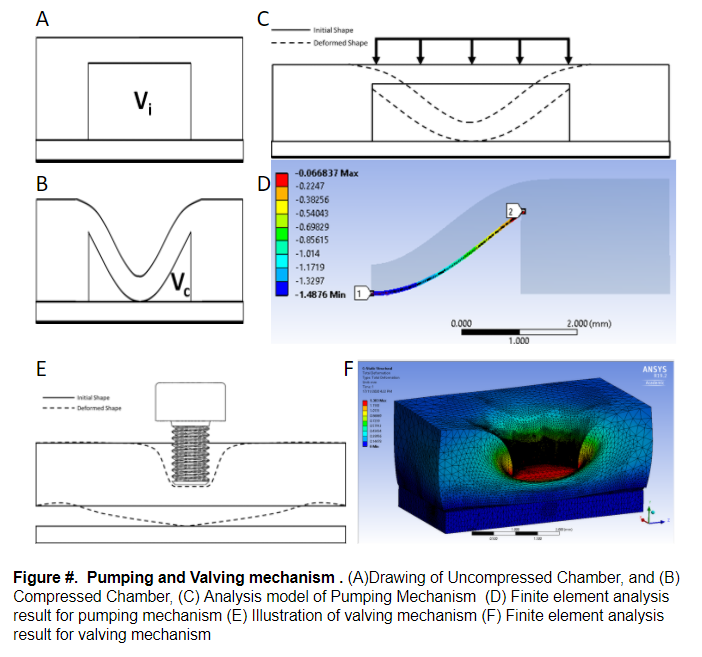
**

**Figure S1.** Drawings of the chamber’s (A) initial volume (V_i_), (B) compressed volume (V_c_) and (C) loading condition. (D) Diagram of the finite element analysis result.

**Figure S2.** Schematics of the electronics

**Table S1: Respiratory Panel 2 (RP2) Controls**

| **Virus/Organism** | **Strain** | **Control 1** | **Control 2** |
| --- | --- | --- | --- |
| Adenovirus Type 1 | N/A | Positive | Negative |
| Adenovirus Type 3 | N/A | Positive | Negative |
| Adenovirus Type 31 | N/A | Positive | Negative |
| C. pneumoniae | CWL-029 | Positive | Negative |
| Influenza A 2009 H1N1 | A/NY/02/2009 | Positive | Negative |
| Influenza A H3N2 | A/Brisbane/10/07 | Positive | Negative |
| Human Metapneumovirus Type 8 | Peru6-2003 | Positive | Negative |
| M. pneumoniae | M129 | Positive | Negative |
| Parainfluenza Type 1 | N/A | Positive | Negative |
| Parainfluenza Type 4 | N/A | Positive | Negative |
| Rhinovirus Type 1A | N/A | Positive | Negative |
| B. parapertussis | A747 | Negative | Positive |
| B. pertussis | A639 | Negative | Positive |
| Coronavirus 229E | N/A | Negative | Positive |
| Coronavirus HKU-1 | Recombinant | Negative | Positive |
| Coronavirus NL63 | N/A | Negative | Positive |
| Coronavirus OC43 | N/A | Negative | Positive |
| Influenza A H1N1 | A/New Cal/20/99 | Negative | Positive |
| Influenza B | B/Florida/02/06 | Negative | Positive |
| Parainfluenza Type 2 | N/A | Negative | Positive |
| Parainfluenza Type 3 | N/A | Negative | Positive |
| RSV Type A | 2006 Isolate | Negative | Positive |

**Table** **S2.** Off the shelf (OTS) component list use in the portable and battery-operated reusable control unit.

| **OTS components** | **Vendor** | **Cost**  **(AUD)** |
| --- | --- | --- |
| Arduino Uno | https://au.rs-online.com/web/p/arduino/7154081 | 31.41 |
| Micro Heaters | https://www.ebay.com/itm/100-550-1020-High-Temperature-MCH-Micro-Ceramic-Heater-Heating-Element-Tablet-/173928724748 | 14.00 |
| Sensors | https://au.rs-online.com/web/p/temperature-sensors-humidity-sensors/0403620/ | 0.80 |
| Resistors | https://au.rs-online.com/web/c/passive-components/fixed-resistors/through-hole-fixed-resistors/ | 2.00 |
| MOSFET | https://www.jaycar.com.au/dual-n-channel-30v-mosfet/p/ZK8821 | 5.50 |
| SPDT Micro Slide Switch | https://www.jaycar.com.au/spdt-micro-slide-switch/p/SS0834 | 2.00 |
| Bi-colour LED | https://au.rs-online.com/web/p/leds/2285685/ | 1.50 |
| Blue LED | https://au.rs-online.com/web/p/leds/2471561/ | 2.00 |
| SPST Micro Tactile Switch | https://au.rs-online.com/web/p/tactile-switches/1613779 | 0.10 |
| PC Mount DC Socket with SPST Rocker Switch | https://www.jaycar.com.au/2-1mm-pc-mount-dc-socket-with-spst-rocker-switch/p/PS0531 | 4.00 |
| Piezo Buzzer | https://au.rs-online.com/web/p/piezo-buzzer-components/0457065/ | 1.40 |
| Prototype Board | https://au.element14.com/multicomp/mc01009/prototype-board-phenolic-72mm/dp/2768279?ost=2768279 | 1.00 |
| **Total Cost (AUD)** | | **$65.71** |
