# Supplementary material for: An ultra-portable, self-contained point-of-care nucleic acid amplification test for diagnosis of active COVID-19 infection
Source: Sci Rep. 2021 Jul 26;11:15176. doi: 10.1038/s41598-021-94652-0 (PMC8313664; doi:10.1038/s41598-021-94652-0)
Supplement: Supplementary file 2 — Supplementary Information 2. [file 41598_2021_94652_MOESM2_ESM.docx]

Title: An ultra-portable, self-contained point-of-care nucleic acid amplification test for diagnosis of active COVID-19 infection

Authors: Hao Deng, ^⸸^Asanka Jayawardena, ^†^ Jianxiong Chan, ^†^ Sher Maine Tan,^†^ Tuncay Alan, ^,⸸^ Patrick Kwan ^,†^

^†^Department of Neuroscience, Central Clinical School, Monash University, Melbourne, VIC 3004, Australia

^⸸^Department of Mechanical and Aerospace Engineering, Monash University, Clayton, VIC 3800, Australia

**Video S1:**Video recording demonstrating the operation procedure of the device, and displaying test result for positive sample (300 copies/µl of virus RNA in water) and negative control (water only)

**Video S2:**Video recording for the thermal profiling of the lysis process at Chamber 1. Thermal profiling was performed by using FLIR i7 infrared thermal camera (sensitivity ± 0.1°C).  Temperature profile showed desired temperature 95 °C maintained for 5 minutes.

**Video S3:**Video recording for the thermal profiling of the LAMP reaction at Chamber 2. Thermal profiling was performed by using FLIR i7 infrared thermal camera (sensitivity ± 0.1°C).  Temperature profile showed desired temperature 60 °C maintained for 30 minutes.
